# Eosinophils Reduce Chronic Inflammation in Adipose Tissue by Secreting Th2 Cytokines and Promoting M2 Macrophages Polarization

**DOI:** 10.1155/2015/565760

**Published:** 2015-11-25

**Authors:** Yi Zhang, Peng Yang, Ran Cui, Manna Zhang, Hong Li, Chunhua Qian, Chunjun Sheng, Shen Qu, Le Bu

**Affiliations:** Shanghai Tenth People's Hospital, Tongji University, Shanghai 200072, China

## Abstract

Obesity is now recognized as a low-grade, chronic inflammatory disease that is linked to a myriad of disorders including cardiovascular diseases, type 2 diabetes, and liver diseases. Recently it is found that eosinophils accelerate alternative activation macrophage (AAM) polarization by secreting Th2 type cytokines such as interleukin-4 and interleukin-13, thereby reducing metainflammation in adipose tissue. In this review, we focused on the role of eosinophils in regulating metabolic homeostasis and obesity.

## 1. Introduction

Obesity, independently associated with a myriad of health conditions including type 2 diabetes, cardiovascular diseases, dyslipidemia, and fatty liver [1, 2], is recognized as the potential threat to human health. As per 2008, WHO official statistics suggested that 1.4 billion population were overweight, of which nearly 300 million were clinically obese adults. In addition, there were nearly 40 million overweight children worldwide [3]. Obesity, especially visceral obesity, plays a vital role in driving the development of insulin resistance (IR) which is a common soil for diabetes, hypertension, and hyperlipidemia [4]. Although extensive studies have been performed in the past decades with an attempt to decipher the molecular and cellular pathogenesis of obesity related insulin resistance, its exact mechanism remains largely elusive, and accordingly we still lack the therapeutic strategies for effective management of obesity associated metabolic disorders.

Over the past 2 decades it has become well established that obesity is associated with a state of chronic low-grade inflammation termed “metainflammation” [5]. The obesity related chronic inflammation is collectively regulated by a variety of immune cells including macrophages, T cells, natural killer cells, and eosinophils. In fact, recent studies have found that eosinophils promote alternative activation macrophage polarization by secreting Th2 type cytokines such as IL-4 and IL-13, consequently leading to reduced metainflammation in adipose tissue, and meanwhile resulting in increased energy expenditure through nonshivering thermogenesis. These studies provide strong evidence demonstrating eosinophils play an unexpected role in regulating metabolic homeostasis through maintenance of adipose AAMs.

## 2. Macrophage Versatility

Mounting evidence has shown that macrophages are key mediators of obesity-induced IR. Indeed, one of the features of metabolically unhealthy obesity is adipose infiltration of macrophages which are derived from differentiation of circulating monocytes and proliferation of local resident macrophages. Upon pathological or physiological stimuli, the circulating monocytes can be differentiated into two types of macrophages: M1 (Classically Activated Macrophage (CAM)) and M2 (Alternative Activated Macrophage (AAM)) [6]. CAMs accumulate in parallel to adiposity in obesity and release cytokines such as IL-1*β*, IL-6, and TNF-*α* creating a proinflammatory microenvironment that represses adipocyte insulin signal transduction, directly contributing to the development of IR and type 2 diabetes mellitus. Conversely, AAMs dominate insulin-sensitive adipose tissue in the lean, which has an important role in regulating metabolism and energy balance and maintaining temperature through nontrembling thermogenesis.

The process of AAMs differentiation is complicated and can be influenced by various endogenous and exogenous factors. The initial step for monocytes migration into fat depots is mediated by chemokine receptor CCR2, which recruits sufficient amount of monocytes and subsequently exposes these cells to a network of cytokine stimulation. In this context, the IL-4/13 pathway plays a dominant role in inducing and promoting M2 polarization of macrophages through the IL-4 receptor (IL-4R). IL-4R can be divided into two categories, type I (IL-4R*α*/*γ*c) and type II (IL-4R*α*/IL-13R*α*1) [7]. At the molecular level, IL-4 binds to the type I receptor with a high affinity, initiating the signal transduction through the IL-4 receptor-associated Janus kinases, giving rise to rapid tyrosine phosphorylation of STAT6. Phosphorylated STAT6 will form a dimmer and subsequently translocate into the nucleus to elicit expression of the lipid-sensing nuclear factors PPAR-*γ* and PPAR-*δ* and their coactivator, the PPAR-*γ* coactivator-1*β* (PGC-1*β*). Importantly, the downstream molecule PGC-1*β* in turn serves as a coactivator for the STAT6 dimer, consequently leading to amplification of the expression of M2-defining markers, such as CD206 and arginase-1 [8–11] and ultimately promoting AAM polarization [12]. It should be noted that the IL-4/13 pathway is critical in enhancing PPAR-*γ* activity, although the transcription of this anti-inflammatory nuclear receptor can be stimulated by various signals such as lipopolysaccharides and transforming growth factor-*β* [9].

## 3. Nonshivering Thermogenesis in Brown Adipose and Catecholamines in AAM

### 3.1. Nonshivering Thermogenesis in Brown Adipose

Normally mature lipid-laden adipocytes are believed to account for only 20%–40% of the cellular content of fat pad, and the others are immune cells and fibroblast [13]. It is becoming more and more clear that the adipose tissue is not only a mere storage depot for energy but also an endocrine and immunologically active organ [14]. In a cold environment, maintenance of body temperature is achieved through a combination of shivering and nonshivering thermogenesis. Adipocytes have been generally divided into two types: white adipocytes that store energy and brown adipocytes where nonshivering thermogenesis occurs. Brown adipocytes generate heat from the metabolism of fatty acids by virtue of their ability to express the mitochondrial uncoupling protein 1 (UCP1) [15]. Recent studies provide compelling evidences showing that the nonshivering thermogenesis in brown adipocytes is closely related to macrophage activation. Moreover, an increasing amount of evidence supports brown adipose tissue (BAT) playing an important role in whole-body glucose homeostasis and insulin sensitization in humans [16, 17]. In addition to energy storage and heat production, we found that adipose tissue presents a chronic low-grade inflammation in diet-induced obese body [18].

### 3.2. Catecholamines in AAMs

Although traditional view holds that maintaining body temperature is primarily controlled by the sympathetic nerve system, recent studies provide strong evidence demonstrating brown fat thermogenesis can also be regulated profoundly by catecholamines released from local AAMs. Exposure to cold rapidly promotes alternative activation of AAMs which secrete catecholamines to induce thermogenic gene expression in brown adipose and triggers lipolysis in white adipose via the *β*3 adrenergic receptors. AAM expresses high levels of arginase-1, CD301, and the mannose receptor (CD206) and secretes anti-inflammatory cytokines including IL-10 and IL-1 receptor antagonist (IL-1Ra) and also has a positive impact on inflammation of adipose tissue resulting in insulin resistance [14, 19].

Catecholamine production by alternatively activated macrophages has been shown to be important for induction of thermogenic and fatty acid *β*-oxidation genes in WAT and BAT of low temperature exposed mice [20]. Signals triggered by the IL-4 pathway induce expression of tyrosine hydroxylase, dopamine decarboxylase, and *β*-hydroxylase which are responsible for synthesis of catecholamine in AAM. Tyrosine hydroxylase is the rate-limiting step in the synthesis of catecholamines [21]. In agreement with these studies, treatment of wild-type macrophages with *α*-methyltyrosine, a specific inhibitor of tyrosine hydroxylase [22], inhibited secretion of noradrenaline into the culture medium and abrogated its lipolytic activity on cultured adipocytes [20]. These findings provide direct evidence that actions of alternatively activated macrophages in BAT and WAT orchestrate the metabolic programs that constitute adaptive thermogenesis. Taken together, in addition to the classical sympathetic nerve system, AAMs constitute a second, parallel circuit for controlling nonshivering thermogenesis.

## 4. Anti-Inflammatory Effects of AAM

### 4.1. Inflammation and Insulin Resistance

It was in the early 1990s that the evidence for a causative role for inflammation in development of obesity related IR became established. In a landmark study published in 1993, Hotamisligil et al. observed that the proinflammatory cytokine Tumor Necrosis Factor-*α* (TNF-*α*) was elevated in circulation and especially in the white adipose tissue of obese rodents [23]. Consistent with this elegant early finding, subsequent studies from multiple independent groups concluded that proinflammatory adipokines and cytokines secreted by the adipose tissue can work on a panel of metabolically active organs such as liver and skeletal muscles to induce insulin resistance and cause glucose intolerance [24].

### 4.2. AAMs Produce IL-10 to Protect against Inflammation

AAMs play pivotal roles in wound healing and immune regulation. This group of unique immune regulatory cells have been further divided into three subgroups according to the differences in their mode of activation. Wound healing M2a macrophages are primarily induced by IL-4 and/or IL-13. They produce anti-inflammatory IL-10, IL-1 receptor antagonist, and arginase. In vitro experiments have shown that macrophages treated with IL-4 and IL-13 do not produce proinflammatory cytokines and thus are less effective than M1 macrophages in killing the invasive pathogens and initiating a proinflammatory response [25]. However, these cells produce polyamines, a component of the extracellular matrix indicative of their major role in wound healing. M2b and c-polarization macrophages are regulatory macrophages distinguished by their method of activation. M2b macrophages are induced through the combined action of TLR and another immune complex or stimuli. These M2b macrophages are able to produce high levels of IL-10 to block the proinflammatory action of IL-12, thus dampening the inflammation [26]. M2c macrophages are induced by IL-10 and express substantial mannose receptor, the cellular surface marker implicated in tissue remodeling [27]. Thus AAMs have a very positive effect in alleviating metainflammation in treatment of obesity and obesity related IR [28].

## 5. Eosinophils Secrete and Raise IL-4 Levels

Eosinophils are associated with immunity and allergy towards helminths, often in conjunction with AAMs. As an important cytokine to activate M2 macrophages, IL-4 has a significant impact on gene expression for heat production, metabolism of fatty acids, and energy output. Due to its critical role in regulating the immune system and energy metabolism, IL-4 has been attracting increasing attention. Using white adipose tissue surrounding gonads of mice with green fluorescent protein gene markers, Wu et al. traced the source of IL-4. After analysis of overnight incubated cells migrating out of minced adipose tissue, they found that a large number of fluorescent cells were eosinophils, while a small amount was CD4 T cells. SVF (stromal/vascular fraction) extracted from the adipose tissue shows eosinophils accounted for 90% of all IL-4-expressing cells [14]. It is therefore inferred that eosinophils are the major source of adipose IL-4. Subsequent investigations elegantly unraveled that the production of eosinophils in bone marrow and their recruitment into white adipose tissue are largely controlled by IL-5 [29, 30]. The main source of adipose IL-5 is a newly recognized population, innate lymphoid type 2 cells (ILC2s), which promotes the accumulation of eosinophils and AAM [30, 31], and loss of this population of immune cells in mice model exacerbated diet-induced obesity and metabolic dysfunction [32]. Further, IL-33, a cytokine previously shown to promote cytokine production by ILC2s, leads to rapid ILC2-dependent increases in visceral adipose tissue (VAT) eosinophils and AAMs [30, 31]. Overall, eosinophils and ILC2s function to attenuate inflammation and thereby restore insulin sensitivity [14, 30]. Additionally, CD4^+^Foxp3^+^ regulatory T cells (Tregs) in VAT have been suggested to be a critical negative regulator in modulating fat tissue inflammation and obesity associated metabolic disorders [33, 34]. In contrast, many other immune cells such as neutrophils, mast cells, B lymphocytes, and various classes of T lymphocytes are all increased in abundance in the obese fat pad, while these cells mainly contribute to impaired insulin sensitivity [35].

Qiu et al. established wild-type mice and IL-4/13 deficient mice model housed at thermoneutrality (30°C), 22°C, or 5°C for 48 hrs and subsequently measured expression levels of marker genes using RT-PCR. Under the conditions of prolonged cold exposure at 5°C, transcription levels of* Ucp-1*,* Pgc1α*,* Cox8β*,* Cidea*,* Elovl3*, and* Cpt1β* in the subcutaneous white fat were all markedly increased, while their increases were greatly diminished by IL-4/13 deficiency. Similarly, wild-type mice maintained at 22°C and 5°C showed dramatically increased UCP-1 protein levels in beige and brown fat depots while this phenotypic change was completely blocked by genetic disruption of the IL-4/13 pathway [36]. The authors of this study further demonstrated that eosinophils were the major IL-4-expressing cells and played an unexpected role in regulating metabolic homeostasis through maintenance of adipose AAMs. These findings indicate that modulating the amount and function of adipose eosinophils via pharmacological approaches may provide an exciting and novel therapeutic strategy in treating human metabolic disorders.

## 6. Metrnl

Metrnl (Meteorin-like) is a myokine that contributes to the phenotype of Pgc1a4-transgenic mice. The Metrnl gene is highly expressed in both muscle and fat and can be induced either by resisting exercise in skeletal muscle or by cold exposure to adipose tissue. Overexpression of Metrnl in mice increases energy expenditure, promotes thermogenic gene expression, and increases the abundance of beige adipocytes. Similarly, administration of recombinant Metrnl to mice recapitulates these phenotypic changes by stimulating thermogenic gene expression, which is accompanied by weight loss in a diet-induced model of obesity [15].

In the course of elucidating the mechanism of Metrnl action, Rao et al. [7] noted that recombinant Metrnl does not act directly on adipocytes or macrophages; however, its effects in vivo are dependent on the IL-4/13 signaling cascade of alternately activated macrophages. Fluorescence-activated cell sorting (FACS) analysis of adipose tissue reveals increased numbers of eosinophils in mice overexpressing Metrnl. The authors subsequently demonstrated that eosinophils indeed are required for Metrnl-induced browning. Finally, a neutralizing antibody raised against Metrnl prevents the accumulation of eosinophils in adipose tissue and reduces the expression of genes associated with alternative macrophage activation and thermogenesis, thereby implicating Metrnl in the physiological browning response as an upstream path of eosinophils for the entire cellular pathways [37].

## 7. Discussion

Although it has become increasingly clear that eosinophils play an important role in regulating energy metabolism via secreting Th2 cytokines and promoting M2 polarization of macrophages, a few important questions still remain unsolved. For instance, the relationship between the cause and consequence of obesity and chronic inflammation remains largely elusive. If the adipose chronic inflammation observed under obese state is secondary to excess energy storage, it would be important to elucidate at the molecular level how excess lipid sequestration in individual adipocytes leads to initiation of this vicious immune response. Additionally, it remains unclear whether eosinophils have a direct activity in alleviating adipose chronic inflammation or the alleviated inflammation in fat pads is a result of elevated energy expenditure and it is related with weight loss.

Secondly, we have not thoroughly studied Metrnl. Many questions remain unanswered. For example, what is the cellular target for Metrnl? Is it on eosinophils? We know that Metrnl, as nerve growth factor, has a significant effect on the treatment of nervous system disorders, particularly hearing loss, Meniere's disease [38], but there is no definitive experimental evidence about Metrnl and IL-4 for the treatment of obesity. It is unclear if local administrations of these factors are capable of generating a systemic effect in treating metabolic diseases. Although some recent studies proposed that increasing eosinophils in adipose tissues induced by parasites can produce beneficial effects in improving glucose tolerance, it is unclear whether this approach can trigger lipid mobilization in fat pads which is critical for weight loss. Nevertheless, recent findings in the field laid a foundation for potential clinical application of IL-4 and Metrnl to treat metabolic diseases such as obesity and diabetes in humans.

## References

[1] Kahn S. E., Hull R. L., Utzschneider K. M. (2006). Mechanisms linking obesity to insulin resistance and type 2 diabetes. *Nature*.

[2] Masuoka H. C., Chalasani N. (2013). Nonalcoholic fatty liver disease: an emerging threat to obese and diabetic individuals. *Annals of the New York Academy of Sciences*.

[3] Flegal K. M., Carroll D., Kit B. K., Ogden C. L. (2012). Prevalence of obesity and trends in the distribution of body mass index among US adults, 1999–2010. *The Journal of the American Medical Association*.

[4] Rask-Madsen C., Kahn C. R. (2012). Tissue-specific insulin signaling, metabolic syndrome, and cardiovascular disease. *Arteriosclerosis, Thrombosis, and Vascular Biology*.

[5] Hotamisligil G. S. (2006). Inflammation and metabolic disorders. *Nature*.

[6] Huh J. Y., Park Y. J., Ham M., Kim J. B. (2014). Crosstalk between adipocytes and immune cells in adipose tissue inflammation and metabolic dysregulation in obesity. *Molecules and Cells*.

[7] Rao R. R., Long J. Z., White J. P. (2014). Meteorin-like is a hormone that regulates immune-adipose interactions to increase beige fat thermogenesis. *Cell*.

[8] Shapiro H., Lutaty A., Ariel A. (2011). Macrophages, meta-inflammation, and immuno-metabolism. *TheScientificWorldJOURNAL*.

[9] Szanto A., Balint B. L., Nagy Z. S. (2010). STAT6 transcription factor is a facilitator of the nuclear receptor PPAR*γ*-regulated gene expression in macrophages and dendritic cells. *Immunity*.

[10] Morris D. L., Singer K., Lumeng C. N. (2011). Adipose tissue macrophages: phenotypic plasticity and diversity in lean and obese states. *Current Opinion in Clinical Nutrition and Metabolic Care*.

[11] Chawla A. (2010). Control of macrophage activation and function by PPARs. *Circulation Research*.

[12] Odegaard J. I., Chawla A. (2011). Alternative macrophage activation and metabolism. *Annual Review of Pathology: Mechanisms of Disease*.

[13] Kanneganti T.-D., Dixit V. D. (2012). Immunological complications of obesity. *Nature Immunology*.

[14] Wu D., Molofsky A. B., Liang H.-E. (2011). Eosinophils sustain adipose alternatively activated macrophages associated with glucose homeostasis. *Science*.

[15] Lee S. D., Tontonoz P. (2014). Eosinophils in fat: pink is the new brown. *Cell*.

[16] Chondronikola M., Volpi E., Børsheim E. (2014). Brown adipose tissue improves whole-body glucose homeostasis and insulin sensitivity in humans. *Diabetes*.

[17] Betz M. J., Enerbäck S. (2015). Human brown adipose tissue: what we have learned so far. *Diabetes*.

[18] Rosen E. D., Spiegelman B. M. (2014). What we talk about when we talk about fat. *Cell*.

[19] Maizels R. M., Allen J. E. (2011). Eosinophils forestall obesity. *Science*.

[20] Nguyen K. D., Qiu Y., Cui X. (2011). Alternatively activated macrophages produce catecholamines to sustain adaptive thermogenesis. *Nature*.

[21] Zhou Q.-Y., Quaife C. J., Palmiter R. D. (1995). Targeted disruption of the tyrosine hydroxylase gene reveals that catecholamines are required for mouse fetal development. *Nature*.

[22] Flierl M. A., Rittirsch D., Nadeau B. A. (2007). Phagocyte-derived catecholamines enhance acute inflammatory injury. *Nature*.

[23] Hotamisligil G. S., Shargill N. S., Spiegelman B. M. (1993). Adipose expression of tumor necrosis factor-*α*: direct role in obesity-linked insulin resistance. *Science*.

[24] Kammoun H. L., Kraakman M. J., Febbraio M. A. (2014). Adipose tissue inflammation in glucose metabolism. *Reviews in Endocrine and Metabolic Disorders*.

[25] Edwards J. P., Zhang X., Frauwirth K. A., Mosser D. M. (2006). Biochemical and functional characterization of three activated macrophage populations. *Journal of Leukocyte Biology*.

[26] Anderson C. F., Mosser D. M. (2002). Cutting edge: biasing immune responses by directing antigen to macrophage Fc*γ* receptors. *The Journal of Immunology*.

[27] Mantovani A., Sica A., Sozzani S., Allavena P., Vecchi A., Locati M. (2004). The chemokine system in diverse forms of macrophage activation and polarization. *Trends in Immunology*.

[28] Harford K. A., Reynolds C. M., McGillicuddy F. C., Roche H. M. (2011). Fats, inflammation and insulin resistance: insights to the role of macrophage and T-cell accumulation in adipose tissue. *Proceedings of the Nutrition Society*.

[29] Mould A. W., Matthaei K. I., Young I. G., Foster P. S. (1997). Relationship between interleukin-5 and eotaxin in regulating blood and tissue eosinophilia in mice. *The Journal of Clinical Investigation*.

[30] Molofsky A. B., Nussbaum J. C., Liang H.-E. (2013). Innate lymphoid type 2 cells sustain visceral adipose tissue eosinophils and alternatively activated macrophages. *The Journal of Experimental Medicine*.

[31] Brestoff J. R., Kim B. S., Saenz S. A. (2015). Group 2 innate lymphoid cells promote beiging of white adipose tissue and limit obesity. *Nature*.

[32] Exley M. A., Hand L., O'Shea D., Lynch L. (2014). Interplay between the immune system and adipose tissue in obesity. *Journal of Endocrinology*.

[33] Chen X., Wu Y., Wang L. (2013). Fat-resident Tregs: an emerging guard protecting from obesity-associated metabolic disorders. *Obesity Reviews*.

[34] Lee B.-C., Lee J. (2014). Cellular and molecular players in adipose tissue inflammation in the development of obesity-induced insulin resistance. *Biochimica et Biophysica Acta—Molecular Basis of Disease*.

[35] Mathis D. (2013). Immunological goings-on in visceral adipose tissue. *Cell Metabolism*.

[36] Qiu Y., Nguyen K. D., Odegaard J. I. (2014). Eosinophils and type 2 cytokine signaling in macrophages orchestrate development of functional beige fat. *Cell*.

[37] Berndt J. D. (2014). The IL-4 brown out. *Science Signaling*.

[38] Jørgensen J. R., Fjord-Larsen L., Wahlberg L. U. Therapeutic use of a growth factor, METRNL.

